# New insights on the development of fungal vaccines: from immunity to recent challenges

**DOI:** 10.1590/0074-02760150335

**Published:** 2015-12

**Authors:** Natasha P Medici, Maurizio Del Poeta

**Affiliations:** Stony Brook University, Department of Molecular Genetics and Microbiology, Stony Brook, NY, USA

**Keywords:** fungi, vaccine, immunity, yeast, strategy, infection

## Abstract

Fungal infections are emerging as a major problem in part due to high mortality associated with systemic infections, especially in the case of immunocompromised patients. With the development of new treatments for diseases such as cancer and the acquired immune deficiency syndrome pandemic, the number of immunosuppressed patients has increased and, as a consequence, also the number of invasive fungal infections has increased. Several studies have proposed new strategies for the development of effective fungal vaccines. In addition, better understanding of how the immune system works against fungal pathogens has improved the further development of these new vaccination strategies. As a result, some fungal vaccines have advanced through clinical trials. However, there are still many challenges that prevent the clinical development of fungal vaccines that can efficiently immunise subjects at risk of developing invasive fungal infections. In this review, we will discuss these new vaccination strategies and the challenges that they present. In the future with proper investments, fungal vaccines may soon become a reality.

In recent years, several studies in the field of medical mycology have been focused on the development of new vaccines against fungal pathogens. Many pertinent reviews and papers have been published with both new strategies and challenges to the development of antifungal vaccines (Deepe Jr 1997, Casadevall et al. 2002, Torosantucci et al. 2005, Cassone 2008, Edwards Jr 2012, Iannitti et al. 2012, de Amorim et al. 2013, Muñoz et al. 2014, Assis-Marques et al. 2015, de Almeida et al. 2015). This increase in interest is due to the rise of dangerous systemic fungal infections, especially related to immunocompromised patients, premature infants, cancer patients and those with invasive treatments for long periods in hospital settings, which are known as high-risk groups (Spellberg 2011, Iannitti et al. 2012, Roy & Klein 2012). High-risk groups in the past decades have been expanding in number owing to advances in the medical field, where new treatments to critical diseases, such as cancer, have arisen (Das & Ranganathan 2012). These treatments improve patient’s survival rates, but can also affect natural barriers of the body or even significantly impact the competence of the immune system of the individual, contributing to an increased vulnerability to infections caused by fungi (Paramythiotou et al. 2014). It is estimated that patients undergoing treatment for haematologic malignancies, such as leukaemia, have a mortality rate of 35% due to systemic fungal infections (Bhatt et al. 2011) while human immunodeficiency virus (HIV)/acquired immune deficiency syndrome (AIDS) patients are significantly affected by opportunistic fungi as *Cryptococcus neoformans*, accounting for 650,000 deaths/year (Del Poeta & Casadevall 2012). By virtue of these facts, it is important to develop vaccines that can protect both immunocompetent and immunocompromised hosts and generate long term immunological memory, using combined mechanisms of innate and adaptive immune response (Roy & Klein 2012). This review seeks to provide an update on the progress made in host-fungi interactions as it relates to vaccine development. This review will cover how the immune system works against fungal infections, the importance of the development of new strategies, the efforts made and challenges that still need to be solved for the advance in this area of fungal vaccines.

## Fungi and the relation with the host

Humans are constantly exposed to many species of fungi; those that can survive at human body temperature can establish different interactions - from symbiotic to pathogenic (Iannitti et al. 2012). Some are well-known for their commensal interactions, like *Candida albicans*, where the physical barriers and the adaptive immune system of the healthy host - the epithelium and IgG/IgA - are thought to control the growth and spread of this yeast. This creates a well-defined tolerance between the host and the fungi (Cassone & Cauda 2012). Most others, however, are environmental fungi that can become opportunistic pathogens in immune compromised hosts, like *C. neoformans* (Iannitti et al. 2012), *Aspergillus fumigatus*(Behnsen et al. 2008),*Blastomyces dermatitidis* (Nanjappa & Klein 2014), *Histoplasma capsulatum*(Martin-Iguacel et al. 2014) and*Coccidioides immitis* (Ampel 2005) *.* Infection by these microorganisms occurs when the host fails to control spores or conidia that have been inhaled into the lungs. From there the infection progresses through the bloodstream and into the brain to cause an invasive mycosis with high mortality rates (Rittershaus et al. 2006, Dagenais & Keller 2009, Del Poeta & Casadevall 2012). Recently, the substantial increase in immunocompromised individuals due to the HIV/AIDS pandemic and the development of medical treatment with invasive profiles and immunosuppression have led to a dramatic increase in the incidence of fungal diseases (Cutler et al. 2007, Brown at al. 2012). Despite efforts to avoid secondary infections, such as the development of combined antiretroviral therapy and the use of antifungal agents for prophylaxis in cancer patients (Staber et al. 2007, Armstrong-James et al. 2014, Brown et al. 2014), the immune dysregulation in such cases can be extremely dramatic and even the species known for its equilibrated relation with the host, such as *C. albicans,* can become a life-threatening pathogen (Romani 2011). In this case, it is important to know how the immune system regulates the relation between host and fungi.

## Immune response against fungal infections

Since the kingdom Fungi besets a heterogeneous group of organisms, it is expected that each one will elicit a different immunological response (Cutler et al. 2007). For all pathogens discussed in this review an interconnected innate and adaptive immune response is necessary for the resolution of the infection (Roy & Klein 2012).

*Innate response* - The innate response against fungi is designed to be as efficient as possible and also stimulates several responses mediated by the adaptive immune system (Santamaria et al. 2011). The first lines of defense are physical barriers, like the skin and mucosal epithelial surfaces in the sites of the body that are constantly being exposed to environmental organisms, including sites such as the mouth, the upper airways and the gastrointestinal and genitourinary tract (Borghi et al. 2014). The epithelium also has an important role by actively discriminating commensal fungi, such as *C. albicans*, which occurs in a nonpathogenic and pathogenic form (Dühring et al. 2015). In addition, some specific cells and molecules from the innate immune system play a very important role. The complement system provides recognition and opsonisation of fungi (Romani 2011, Borghi et al. 2014). Opsonisation is extremely important for the phagocytosis of pathogens like*C. neoformans* and its deficiency leads to a higher susceptibility to the disease caused by these fungi (Rohatgi & Pirofski 2015). Defensins play an antifungal role by permeabilising target membranes and are secreted by the epithelium and Paneth cells (Ganz 2003). Collectins are soluble pattern recognition receptors that help in the recognition of fungi, eliciting an inflammatory response against these microorganisms and modulating inflammation by assisting in the opsonisation of the intruder (Cutler et al. 2007, Gupta & Surolia 2007). Phagocytic cells, such as macrophages, dendritic cells (DCs) and neutrophils can quickly recognise fungi through a variety of receptors and combat fungal pathogens by phagocytosis and production of antimicrobial components, like oxygen radicals (Cutler et al. 2007, Roy & Klein 2012, Mueller-Loebnitz et al. 2013) (Blanco & Garcia 2008). Phagocytes can produce cytokines that help in the maturation of T CD4^+^ cells toward different and important subtypes to combat the fungi (Rohatgi & Pirofski 2015). Lastly, DCs are also active against fungal pathogens and are considered the most important connection between the innate and adaptive immune system (Mueller-Loebnitz et al. 2013). These antigen-presenting cells (APCs) can ingest different species of fungi and mature (Cutler et al. 2007) to present those pathogens through major histocompatibility complex (MHC) class I or II and express molecules necessary to fully activate T-helper cells (Roy & Klein 2012). Furthermore, APCs can recognise different structures from fungal cells through nonspecific receptors like Toll-like receptors and dectin receptors. This recognition leads to the production of cytokines that also stimulate phagocytic cells (Mezger et al. 2008, Roy & Klein 2012, Mueller-Loebnitz et al. 2013).

*Adaptive response* - After stimulation of the innate immune system, it is essential that T-cells are activated for a successful elimination and development of protective immunity against fungi (Cutler et al. 2007). Hence, the majority of invasive fungal infections occur in condition of T-cell deficiency. The specific cytokines expressed by APCs cells like DCs and macrophages are crucial for the differentiation of CD4^+^ T-cells [T-helper (Th) cells] (Hamad 2011, LeidbundGut-Landmann et al. 2012, Rohatgi & Pirofski 2015). The different cytokine milieu produced by components of the innate immune system lead to the differentiation of CD4^+^ T-cells towards the Th1 or Th17 subtypes. Once activated, these T-cell subtypes can produce pro-inflammatory cytokines like interferon gamma (IFN-γ), tumour necrosis factor alpha and interleukin (IL) 17/22 (Wüthrich et al. 2012). Those molecules are extremely important for the clearance of the infection, since they recruit neutrophils and help control systemic fungal diseases (van de Veerdonk & Netea 2010, Gibson & Johnston 2014). Furthermore, T-helper cells are known for their importance in the generation, maintenance and differentiation of the other type of T-cells, CD8^+^ (killer T-cells). CD8^+^ T-cells are also produced in the absence of CD4^+^cells and play important roles in immunity especially in the context of diseases in which CD4^+^ cells are deficient (van de Veerdonk & Netea 2010, Nanjappa et al. 2012).

T CD8^+^ cells are cytotoxic T-cells, which possess the ability to kill extracellular and intracellular pathogens, as well as tumourigenic cells, through the release of microbial products, known as granulysins (Oykhman & Mody 2010). They are activated through a different mechanism when compared to T CD4^+^ cells. However, they are found to be just as important as the latter since in their absence, CD8^+^T-cells can be protective (van de Veerdonk & Netea 2010, Verma et al. 2014). This activating mechanism is extremely important when the aim is to generate vaccines that can induce immunity against fungal pathogens in all groups of patients (Verma et al. 2014). Since the majority of systemic fungal infections occur frequently in HIV patients that lack an efficient CD4^+^ T response, the use of pathways that do not require this cell type is a valid alternative (Iannitti et al. 2012, Nanjappa et al. 2012). Recent data has shown that CD8^+^ T-cells can efficiently become long-term memory cells, mediate resistance and maintain their high number and phenotype for a long period after vaccination, even in the absence of T-helper cells (Nanjappa et al. 2012).

Lastly, it has been suggested that humoral immunity contributes to the host defense against fungal infections. Although a lot of controversy still exists when defining the importance of antibodies in the resolution of infection, it has been found that antibodies can target antigens on the fungal cell wall and opsonise these pathogens (Verma et al. 2014). Once bound, antibodies can elicit microbicidal activity and alterations in gene expression in the fungi that modify metabolism and prevent virulence (Cutler et al. 2007, Brena et al. 2011, Verma et al. 2014). Additionally, antibodies can trigger other pathways, such as phagocytosis and the complement system, to aid in the elimination of fungi (Hamad 2011, Wüthrich et al. 2012, Verma et al. 2014). Certain antibodies have direct fungicidal activity by preventing budding and cell growth in vitro. Antibodies against glucosylceramide have been shown to have this direct effect on fungi, suggesting that they can be used as a therapy or in combination with other existent treatments (Rodrigues et al. 2000, 2007).

## Importance of fungal vaccines

As previously described, fungal diseases are rare in immunocompetent individual whereas groups of immunocompromised individuals often are at a risk of developing invasive fungal infections (Spellberg 2011). Some high-risk groups that can be highlighted are HIV patients, cancer patients and those receiving immunosuppressive treatments, such as corticoids (Spellberg 2011, Brown et al. 2012, 2014, Cassone & Cauda 2012). The development of new treatments, especially those aggressive immunosuppressive therapies will continue to rise and consequently increase the number of individuals in the high-risk groups for invasive fungal infections (Spellberg 2011). The impact of the increase in the number of people affected by fungal diseases can be already seen, such as in the case of *C. albicans* whose mortality rate can reach 60% when associated with invasive infection (Moryiama et al. 2014). Additionally, in the United States of America (USA), hospitalisation costs associated with the treatment of candidiasis are estimated at US$ 2-4 billion/year. Invasive candidiasis is particularly costly due to the longer treatment stay when compared to other infections (Wilson et al. 2002, Hidron et al. 2008, Spellberg 2011, Moryiama et al. 2014). Since it has been proven that immune and mucosal damage are required for *Candida* dissemination (Koh et al. 2008), it is crucial to protect patients under these conditions. Another example is the yeast *C. neoformans*, an environmental fungus causing the most common fungal meningoencephalitis worldwide in immunocompromised patients (Rittershaus et al. 2006). Infections caused by this fungus account for more than 600,000 deaths per year which is statistically significant when compared to the era prior to the mid-1950’s, where cases were not more than 300/year. Also, it has been found that other *Cryptococcus* species, such as *Cryptococcus gattii*, can also affect immunocompetent hosts (Kidd et al. 2004, Del Poeta & Casadevall 2012, Espinel-Ingroff & Kidd 2015, LIFE 2015, Rella et al. 2015). Finally, species from the genus *Aspergillus* are associated with the second most common cause of nosocomial infection in the USA (Spellberg 2011, Bourgeois & Kuchler 2012, Vermeulen et al. 2014). The mortality rates for invasive aspergillosis can reach 80% in some cases, which is even more dramatic than candidiasis (Perlroth et al. 2007). It is estimated that at least three million people are affected by chronic pulmonary aspergillosis worldwide (LIFE 2015).

Most of these infections afflict patients with severe immunodeficiency. In addition, current antifungal drugs have limitations such as toxicity, availability, spectrum of activity and may have major drug-interactions. There is also a problem with the development of resistance when used for long periods of time (Denning & Bromley 2015). Based on these limitations, it is important to develop new strategies involving antifungal vaccines in order to reduce the risk of death of these patients (Iannitti et al. 2012). Currently, a considerable number of research groups have been focusing on the creation of new fungal vaccines that can generate long-term memory and can be used in all groups, from high-risk to healthy patients and improve their quality of life (Spellberg 2011, Cassone & Casadevall 2012). The fact that fungal pathogens afflict primarily immunocompromised subjects is a major challenge for the generation of a fungal vaccine, as immunocompetency is often required for the generation of immunity against an infectious disease. Therefore, current research centres on development of vaccines which can be used during immunodeficiency or immediately prior to the development of a severe immunodeficiency.

## Efforts to develop new strategies

The goal of an efficient fungal vaccine is to generate immune responses that will lead to immunological memory and protection against a recurrent exposure to fungi and their conidia/spores (Iannitti et al. 2012). In recent years, many vaccine candidates have been tested against some fungal pathogens (Nanjappa & Klein 2014), such as *C. albicans*, *Aspergillus*spp, *Cryptococcus* spp, *Blastomyces* spp,*Paracoccidioides brasiliensis* and *Sporothrix*spp.

*C. albicans*- Several candidate vaccines have been studied that utilise fungal cell wall polysaccharides, proteins and/or live attenuated strains as strategies for *Candida* vaccines (Wang et al. 2015). Also, different strategies to enhance the activity of the vaccines have been published, including adjuvants and delivery systems (Edwards Jr 2012, Portuondo et al. 2015). All this effort is resulting in promising new discoveries to combat this fungus*.*

In 2012, Schmidt et al. published new work that utilised the N-terminal portion of the agglutinin like sequence 3 protein (Als3p) as a vaccine. To enhance antigenicity, after the production of the protein using *Saccharomyces cerevisiae* expressing cell line, the protein was purified and formulated with aluminium hydroxide as an adjuvant. Once tested in mice and nonhuman primates, the vaccine was tested in healthy humans. The vaccination occurred in two doses, in ascending concentrations and with placebo as control. Seventy-three adults, ranging from 19-47 years old, were tested and the results showed interesting outcomes. All subjects had a rapid response and generated anti-Als3p antibody after the first dose, including those that did not have detectable antibodies against this protein prior to the vaccination. The second dose elicited a very similar IgG response to the first one; however the IgA1 response was increased. T-cell responses were measured by the presence of cytokines like IL-17 and IFN-g and it was found that the higher dose was the most efficient, generating a robust T-cell response independent of antibodies. The vaccine, however, was not tested in patients under treatment of corticosteroids and antibiotics, the main risk groups affected by*Candida.* Nonetheless, based on the results this is a promising candidate, especially because it showed positive protection against disseminated*Candida* and vaginitis caused by this pathogen.

Also in 2012, de Bernardis et al. published the development of another protein vaccine, however, utilising a recombinant version of the secreted aspartyl proteinase 2 as the antigen in the vaccine with a virosome as adjuvant. Saps are important virulence factors from *Candida* and play important roles in vaginitis (Cassone 2014). The subjects were mice, which were vaccinated by intravaginal route. The results showed generation of specific protective antibodies against the protein that also cross reacted with different Saps. The vaccine showed to be low in toxicity and could be used in human tests. The clinical trials have already started with the vaccine being delivered by intramuscular and intravaginal routes, but the results have not been released to the public domain thus far (Edwards Jr 2012).

Other strategies have been previously addressed which include the use of an engineered live attenuated strain of *C. albicans* and the use of components of the cell wall in murine models (Saville et al. 2009, Edwards Jr 2012, Cassone 2014). Those vaccines showed to be efficient, but they have not been tested in humans (Cassone 2014). The live attenuated strategies are particularly challenging, due to the high risk of introducing live organism into a human host and even more so in the case of immunocompromised hosts.

*Aspergillus spp* **-** *A. fumigatus* has not received due attention because only severe immunocompromised patients are typically affected by this fungus and this would make vaccination very difficult (Spellberg 2011). However, we now know that apparent immunocompetent subjects can also be affected by aspergillosis (Taccone et al. 2015). In addition, we also know that certain immunocompromised patients can respond to vaccination (Stevens et al. 2011, Ljungman 2012, Rubin et al. 2014). Based on these facts, some vaccines have been designed to prevent aspergillosis (Stevens et al. 2011).

Pioneer studies in this field used intranasal application of crude*Aspergillus* antigens to generate CD4^+^ Th1 immunity and protect them from pulmonary aspergillosis (Cenci et al. 2000). Importantly, corticosteroid immunosuppressed mice could also respond to a sonicated vaccine in a positive way, generating protection against the disease (Ito & Lyons 2002).

A novel strategy used by Stuehler et al. (2011), was the discovery of the *A. fumigatus* epitope p41 from the cell wall glucanase, named Crf1, as an important immunogenic molecule. In several experiments, they showed that this epitope can be presented through three different MHC class II alleles. It was also shown the production of Th1 cells that can cross-react with *C. albicans*. This was a very important finding, since this epitope could elicit immune response against two very important fungal pathogens in humans.

Recently, a panfungal vaccine using β-glucans of *S. cerevisiae* was shown to generate protection against several pathogenic fungi, including *A. fumigatus*. Interestingly, this vaccine did not need an adjuvant to generate protection. However, the studies were performed in immunocompetent mice and therefore do not indicate if the vaccine would work in condition of immunodeficiency. It is still possible to propose use of this vaccination strategy in immunocompetent subjects, such as those awaiting an organ transplant. Immunity against aspergillosis could be achieved before they become immunocompromised (Liu et al. 2011).

All this effort can lead to future new strategies in the prophylaxis of aspergillosis, which still need a lot of work. However, the development of a panfungal vaccine that protects against this disease may be one of the most promising strategies so far (Liu et al. 2011,Stevens et al. 2011).

*Blastomyces spp, Paracoccidioides spp and Sporothrix spp* **-** Endemic mycoses are diseases caused by fungi present in the nature and seldom are transmitted from human to human (Lorthoraly et al. 1999). The species considered endemic share similar behaviour, are limited to certain geographic locations and, in contrast to the species previously described, can cause invasive fungal infections in healthy hosts more frequently (Kauffman 2006). Species belonging to the genus *Paracoccidioides* spp and *Sporothrix* spp can be considered endemic fungi that frequently cause diseases in Latin American countries like Brazil, Argentina, Colombia and Venezuela (Bagagli et al. 1998, Kauffman 2006, Sbeghen et al., unpublished observations), while fungi belonging to the species *Blastomyces* are endemic in North America, with occasional outbreaks in Africa and Asia (LIFE 2015).

In this area, one important work was completed by Wüthrich et al. (2003), in which they vaccinated T CD4^+^depleted mice with an attenuated strain of *B. dermatitidis* lacking the gene for the adhesin BAD1, indispensable for pathogenesis of this species. The mice were vaccinated two times two weeks apart and challenged with a wild type strain of *B. dermatitidis*. After analysis of the mouse response, it was observed that vaccinated mice could resist the infection for a longer period than unvaccinated mice independent of T CD4^+^ response and maintain persistent immunity. This experiment was important in showing that the host can rely on CD4^+^ T-cell independent immune pathways, which could be an option when vaccinating immunocompromised subjects, such as the ones affected by blastomycosis.

With the increase of diseases caused by *Sporothrix schenkii* and*Sporothrix brasiliensis* in urban areas, scientists have begun to analyse the pathogenicity of these species in order to develop new ways to control these infections (de Almeida et al. 2015). In 2015, de Almeida et al. developed therapeutic antibodies, or passive immunisation, comprised of monoclonal antibodies against the glycoprotein 70 (gp70) from *S. schenkii* that were previously proven to be effective against sporotrichosis caused by this species (Nascimento et al. 2008). After treatment of mice infected with different strains of *S. schenkii* and *S. brasiliensis*, the therapeutic antibodies were shown to decrease fungal burden in mice organs, such as liver and spleen. Since therapeutic antibodies provide passive immunisation and do not induce the generation of long-lasting memory which active immunisations do (Dan & Levitz 2006), they can be used as a treatment option, especially in immunocompromised patients, when the stimulation of active immunity is not possible (Wang et al. 2015). Although a prophylactic vaccine has yet to be developed against *Sporothrix* spp, the gp70 used as antigen in this study was shown to be important in the pathology of these species (de Almeida et al. 2015) and further studies could lead to the development of this protein as a new vaccine.

In the case of paracoccidioidomycosis (PCM), the disease can appear in two clinical forms, acute and chronic. In each case, the treatment requires long, toxic and intensive antifungal therapy with sulphonamides combined with amphotericin B or azoles (Muñoz et al. 2014, Assis-Marques et al. 2015). Despite the long treatment, which can reach up to six months, relapses are frequent (Assis-Marques et al. 2015). In order to develop alternative treatments that can generate host protection, several researchers have been investigating *Paracoccidioides* components for use as vaccines against PCM.

In 2013, de Amorim et al. showed that a modified peptide derived from the antigen gp43 from *P. brasiliensis*, named P10, could protect mice against this pathogen. The mice were vaccinated with a plasmid vector containing the peptide and challenged with the pathogenic strain of the yeast. Vaccination was found to reduce pulmonary fungal burden and resolve the pathological alterations induced by the infection, like the formation of granulomas. The work also found that the vaccine induced the production of T-reg cells, which are involved in the maintenance of immunological memory. Later, the same group also demonstrated the effectiveness of the same vaccine in immunosuppressed mice, which led to the production of Th1 cells predominantly. When combined with the correct fungal chemotherapy treatment, the use of P10 as an adjuvant is a promising strategy for the treatment of PCM and prevention of relapses (Muñoz et al. 2014).

Assis-Marques et al. (2015) developed a mechanism utilising *S. cerevisiae* expressing gp43 as a vehicle for immunisation against *P. brasiliensis*. Their hypothesis was based on the fact that *S. cerevisiae* has components in its cell wall with the ability to elicit a strong immune response and could serve as an ideal adjuvant. After intraperitoneal immunisation, they observed a significant decrease in fungal burden in mice organs after 30 days of immunisation. In addition, several cytokines were detected in lungs and spleen, showing high concentrations of IL-12 and IFN-g. More work needs to be done with this vaccine candidate, especially to prove the production of long lasting immune memory (Assis-Marques et al. 2015).

*Cryptococcus spp* **-** As with aspergillosis, vaccines against *Cryptococcus* spp need to be efficient in patients with severe T-cell deficiency, like HIV/AIDS patients (Spellberg 2011). It is believed that patients are asymptomatic during initial infection by this genus. Once cryptococcosis occurs in an adult with immune defects, it is thought that that the fungus has changed from a latent state to a case of reactivation (Datta & Pirofski 2006). Based on the fact that some *Cryptococcus* spp can cause diseases in both immunocompromised and immunocompetent hosts, a vaccine that can prevent the recurrent disease and the acute form is the ideal solution (Datta & Pirofski 2006).

The first studies in this field comprised the use of an antiphagocytic antigen from the capsule of *C. neoformans*, the glucuronoxylomannann (GMX), as a vaccine (Devi et al. 1991). Since GMX showed low immunogenicity and T-cell independent nature, which is not desired for the vaccination of HIV patients, the vaccine was constructed with tetanus toxoid and generated elevated levels of specific anti-GMX antibodies in mice (Devi 1996). Although the problem of immunogenicity was solved, when administrated in mice, the vaccine was shown to produce nonprotective antibodies (Casadevall & Pirofski 2005, Datta & Pirofski 2006).

In 2011, Wozniack et al. administrated an engineered strain as a vaccine of*C. neoformans* that could express IFN-g into T-cell depleted mice in order to evaluate the generation of protective immunity in the absence of CD4^+^ and/or CD8^+^ T-cells. After vaccination, the mice were challenged with a secondary pulmonary infection using a pathogenic strain. It was shown that protection could be generated in T-cell-deficient hosts, demonstrating that it is possible to generate a protective immune response to*Cryptococcus* even after becoming immunocompromised, like in cases of HIV.

Recently, a study proposed the use of a live attenuated strain as a vaccine (Rella et al. 2015). This mutant lacks the sterol glucosidase enzyme (Δsgl1), leading to a dramatic accumulation of sterol glucosides in the cells. Mice infected with the *Δsgl1* cells were all alive after 90 days and they were able to eliminate the mutant cells from the lung after only 14 days. If these mice were then challenged with virulent*Cryptococcus* strains, either *C. neoformans* or*C. gattii*, they were able to efficiently control the infection. Most interestingly, the administration of *Δsgl1* could elicit a protective immunity also in T CD4^+^ deficient mice. This is very encouraging considering that cryptococcosis is particularly frequent in condition of CD4^+^ T-cell deficiency.

## Challenges and concluding remarks

As previously discussed, several groups have been working on different strategies to advance the field of fungal vaccines. Some of the vaccine candidates have already gone through clinical trials Phase I in humans and are showing good progress towards the development of an efficient method of fungal immunisation (de Bernardis et al. 2012, Schmidt et al. 2012). However, some other candidates are experiencing setbacks due to the combination of several issues (Edwards Jr 2012). For instance, vaccines that utilise live attenuated strains (Saville et al. 2009) can face several safety challenges for use in humans (Edwards Jr 2012). The fact of fungal pathogens affect mostly immunocompromised subjects greatly limits the generation of fungal vaccines. Thus, it is important that fungal vaccines could elicit protection also in immunocompromised subjects, without any risk of aggravation of the underlying disease or/and the development of the fungal disease due to the administration of the vaccine (Cassone 2008, Chatuverdi & Wormley Jr 2013). Potential high costs in preparing the vaccines are a challenge considering that the revenue is obtained from vaccinating only a population at risk of developing fungal infections. In the case of endemic mycosis, a vaccine that can maintain immunity in a small and confined population does not attract enough investment to develop vaccines against these species (Spellberg 2011). Additionally, a vaccine against commensal organisms (e.g., *Candida* spp) could be a challenge as autoimmunity against the commensal fungal organism may become an issue (Fidel & Cutler 2011). Despite these challenges, vaccines against primary fungal pathogens and opportunistic fungi are becoming a reality in clinical trials and the efforts that many research groups have put into developing strategies to produce an efficient antifungal vaccine have proven to be fruitful.

The world population is changing and it is expected that the number of immunocompromised subjects will continue to increase in the future and, as a consequence, fungal infections will continue to rise. As the development of new antifungal drugs is now becoming a priority in academia and industry, we should also invest in the field of fungal vaccines, even if the revenue would be less than those for bacterial and viral vaccines.
